# Editorial: Next-generation dental materials engineered for mineralized tissue reconstruction: advances, challenges and opportunities

**DOI:** 10.3389/fdmed.2025.1618199

**Published:** 2025-05-20

**Authors:** Paulette Spencer, Conrado Aparicio, Candan Tamerler

**Affiliations:** ^1^ Institute for Bioengineering Research, University of Kansas, Lawrence, KS, United States; ^2^ Department of Mechanical Engineering, University of Kansas, Lawrence, KS, United States; ^3^ Bioengineering Program, University of Kansas, Lawrence, KS, United States; ^4^ BOBI-Bioinspired Oral Biomaterials and Interfaces, UPC-Universitat Politènica de Catalunya, Barcelona, Spain; ^5^ Catalan Institute for Research and Advanced Studies (ICREA), Barcelona, Spain; ^6^ SCOI, Study and Control of Oral Infections, Faculty of Odontology, UIC Barcelona-Universitat Internacional de Catalunya, Sant Cugat del Vallès, Spain; ^7^ IBEC, Institute for Bioengineering of Catalonia, Barcelona, Spain

**Keywords:** adhesives, peptide-polymer hybrids, machine learning for peptide design, caries, composites

**Editorial on the Research Topic**
Next-generation dental materials engineered for mineralized tissue reconstruction: advances, challenges and opportunities

Dental caries is one of humankind's most prevalent chronic diseases—untreated dental caries in permanent teeth affects 2.3 billion people across the globe (1). This chronic condition persists throughout an individual's lifetime, and remains an elevated concern for both adults and children (2, 3). Untreated dental caries has severe consequences including unremitting pain, inflammation, the spread of infection to bone and soft tissue, reduced quality of life, missed school and lost productivity (2).

Dental materials play a critical role in caries prevention as well as the repair and reconstruction of lost and damaged mineralized tissues. While a variety of dental materials have been developed, many of the early approaches focused on adapting materials that were originally developed for other applications. Early strategies devoted limited attention to materials that would actively engage mineralized tissue repair processes and the intrinsic complexity of material/tissue interfaces was frequently overlooked (4–6). Material/tissue interfaces are sensitive to a variety of pathogenic and inflammatory reactions as well as a diverse spectrum of mechanical and chemical stresses.

Development of novel materials and innovative approaches is critical to addressing the silent epidemic of dental caries. As the dental profession discontinues the use of the most common dental materials, advancements in areas such as, bioactive, stimuli-responsive materials, and materials that modulate the oral microbiome, have attracted attention. (Boone et al.; 5–13)

The development of regenerative and reconstructive dental materials is a multi-dimensional problem—the materials must provide form, function, esthetics, long-term mineralized tissue repair and favorable clinical outcomes. The aim of this Collection of Publications in the *Frontiers of Dental Medicine* is to explore challenges and opportunities in the development of dental materials that engage mineralized tissue repair processes while also inhibiting interfacial damage provoked by pathogens, mechanical and chemical stresses. This editorial will highlight the diverse research featured in this collection.

Dental composites have become the most popular material for the repair of lost or damaged tooth structure, but composite-restorations can fail to provide long-term function (6, 9). The failure is a multifactorial problem that includes the patient's risk for dental caries, complex microbial communities that organize as biofilms, material and material/tissue interface degradation (7). Dental composite lacks the inherent capability to seal gaps at the interface between the restorative material and tooth structure. The low-viscosity adhesive that infiltrates demineralized dentin to form the hybrid layer and bond the composite to the tooth is intended to seal this interface, but the adhesive seal to dentin is fragile—it is readily damaged by acids, enzymes, and oral fluids. The article titled, Synergistic enhancement of hydrophobic dental adhesives: autonomous strengthening, polymerization kinetics, and hydrolytic resistance Korkmaz et al. describes original research on the structure and properties of a newly formulated methacrylate-based, alkoxysilane-containing polymer (Korkmaz et al.). The polymer resists hydrolysis-mediated degradation by providing intrinsic reinforcement through synergistic stimulation of free-radical polymerization, sol-gel reaction, and hydrophobicity. The polymer shows great promise as a new generation dentin adhesive.

Resin-based composites catalyzed the advancement of minimally invasive approaches. The article titled, Recent advances in direct adhesive restoration resin-based dental materials with remineralizing agents Mai et al. reviews the diverse compounds added to adhesives to remineralize mineral-depleted dental hard tissues (Mai et al.). The adhesives are formulated to address dental-composite restoration failures—failures that are linked to pathogens, oral fluids, enzymes and acids that infiltrate gaps and crevices at the composite/tooth interface. The review describes challenges, opportunities and barriers to the translation of these materials from the laboratory to the clinic.

The article titled, Scale dependent nanomechanical properties of dentin adhesive and adhesive-collagen composite Singh et al. describes original research focused on rigorous investigation of adhesive and adhesive-collagen constructs, i.e., hybrid layer mimics (HL-mimics) (Singh et al.). The nanoscale mechanical properties of model methacrylate-based adhesive and HL-mimics were determined using nanoindentation and nano-dynamic mechanical analyses (DMA). Both the neat adhesive and HL-mimics are heterogeneous, viscoelastic materials at the spatial scales studied in this investigation. The measured frequency dependent storage and loss moduli indicate that both the adhesive and HL-mimics will likely exhibit creep deformation and rate-dependent behavior in physiological function.

Multi-pronged strategies are necessary to address the complex, heterogeneous reactions that occur within the microenvironment of the material/tissue interface. The article titled, Engineering peptide-polymer hybrids for targeted repair and protection of cervical lesions Spencer et al. explores the multi-factorial elements that contribute to root surface lesions and discusses a multi-pronged strategy to both repair and protect root surfaces (Spencer et al.). By 2060, nearly 100 million people in the U.S.A. will be over the age of 65 and one-third of these older adults will have root caries. Nearly 80% of these older adults will have dental erosion. The limitations of current approaches and high prevalence of root caries and dental erosion create an urgent need for microinvasive therapies that can: (a) remineralize damaged dentin; (b) inhibit bacterial activity; and (c) provide durable protection for the root surface. The multi-pronged strategy integrates engineered peptides, novel polymer chemistry, multi-scale structure/property characterization and predictive modeling to develop a durable, microinvasive treatment for root surface lesions.

Our understanding of oral microbial communities guides the exploration of relationships between this community and their impact on the human health-disease balance. The oral microbiome is a highly diverse system; microbial dysbiosis have severe impact on periodontal diseases, and overall health conditions (13, 14). Machine learning can provide valuable insights while also enabling predictive approaches for addressing the health-disease balance. The article titled, Machine learning-enabled design features of antimicrobial peptides selectively targeting peri-implant disease progression Boone et al. is original research focused on the generation of an interpretable, transparent machine learning (ML) model. Peri-implantitis is a complex infectious disease that manifests as progressive loss of alveolar bone around dental implants and hyper-inflammation associated with microbial dysbiosis. The ML model predicted the antimicrobial peptides that can selectively target keystone pathogens in peri-implantitis without harming the commensal species. Targeting keystone pathogens and restoring bacterial community offer a novel therapeutic approach for successful mitigation of disease progression. As promising alternative therapeutics, antimicrobial peptides offer bacterial specificity and targeted inhibitory activity, and transparent ML approaches can enable the prediction of such functions. The approach offers a ML-enabled path for developing antimicrobial peptide-based therapies—therapies that offer promise for restoring oral health by targeting microbial communities impacting disease progression in peri-implantitis.
